# Distinct characteristics and severity of brain magnetic resonance imaging lesions in women and men with multiple sclerosis assessed using verified texture analysis measures

**DOI:** 10.3389/fneur.2023.1213377

**Published:** 2023-08-10

**Authors:** Zahra Hosseinpour, Olayinka Oladosu, Wei-qiao Liu, G. Bruce Pike, V. Wee Yong, Luanne M. Metz, Yunyan Zhang

**Affiliations:** ^1^Department of Biomedical Engineering, Schulich School of Engineering, University of Calgary, Calgary, AB, Canada; ^2^Department of Neuroscience, Faculty of Graduate Studies, University of Calgary, Calgary, AB, Canada; ^3^Department of Clinical Neurosciences, University of Calgary, Calgary, AB, Canada; ^4^Department of Radiology, University of Calgary, Calgary, AB, Canada; ^5^Hotchkiss Brain Institute, Cumming School of Medicine, University of Calgary, Calgary, AB, Canada

**Keywords:** texture analysis, brain MRI, gray-level co-occurrence matrix, percentile thresholding, lesion severity, multiple sclerosis, sex difference

## Abstract

**Background and goal:**

*In vivo* characterization of brain lesion types in multiple sclerosis (MS) has been an ongoing challenge. Based on verified texture analysis measures from clinical magnetic resonance imaging (MRI), this study aimed to develop a method to identify two extremes of brain MS lesions that were approximately severely demyelinated (sDEM) and highly remyelinated (hREM), and compare them in terms of common clinical variables.

**Method:**

Texture analysis used an optimized gray-level co-occurrence matrix (GLCM) method based on FLAIR MRI from 200 relapsing-remitting MS participants. Two top-performing metrics were calculated: texture contrast and dissimilarity. Lesion identification applied a percentile approach according to texture values calculated: ≤ 25 percentile for hREM and ≥75 percentile for sDEM.

**Results:**

The sDEM had a greater total normalized volume yet smaller average size, and worse MRI texture than hREM. In lesion distribution mapping, the two lesion types appeared to overlap largely in location and were present the most in the corpus callosum and periventricular regions. Further, in sDEM, the normalized volume was greater and in hREM, the average size was smaller in men than women. There were no other significant results in clinical variable-associated analyses.

**Conclusion:**

Percentile statistics of competitive MRI texture measures may be a promising method for probing select types of brain MS lesion pathology. Associated findings can provide another useful dimension for improved measurement and monitoring of disease activity in MS. The different characteristics of sDEM and hREM between men and women likely adds new information to the literature, deserving further confirmation.

## 1. Introduction

Multiple sclerosis (MS) is a complex and highly heterogeneous disease with unpredictable outcome. While the exact mechanisms are still unclear, focal lesions remain to be important signatures of MS pathology, especially in the relapsing-remitting subtype (RRMS) (1). Further, the lesions are considerable different even within an individual regarding the type of pathological processes involved, such as demyelination and remyelination (2). Understanding the degree of injury and repair associated with the lesions would be invaluable for optimal disease evaluation and management. However, characterization of lesion severity *in vivo* is challenging, highlighting a critical need for development of new methods.

Magnetic resonance imaging (MRI) is a promising method for assessing neuropathology. Along with data science methods, MRI has shown a considerable potential for identifying lesion characteristics in MS (3–5). Currently much effort is associated with advanced MRI. Based on macro- and micro-scale measures of tissue damage using diffusion MRI, one study differentiated brain MS lesions into two types using a k-means clustering algorithm and showed that lesions with greater diffusion changes correlated with worse clinical outcomes (4). Likewise, using MR Spectroscopy and diffusion imaging, another study evaluated a group of RRMS participants. By dividing brain MS lesions into mild and severe types based on median radial diffusivity (RD), they found that only the mild lesions showed metabolite changes in favor of repair (5). However, advanced MRI do not always outperform conventional MRI measures. In assessing the change of lesions as an indicator of neuroprotection and repair, a study investigated magnetization transfer ratio and mean diffusivity, as well as conventional MRI indices such as signal intensity and T1/T2 ratio, assisted by a percentile categorization approach. Through mixed effects modeling, this study discovered that the 25^th^ percentile (25%^ile^) of normalized proton density-weighted signal intensity had the greatest sensitivity in sample size estimation among all proposed imaging measures (6). Furthermore, advanced MRI is not a part of routine practice in many clinical settings.

Conventional MRI is widely available. While pathologically non-specific in MS (7), conventional MRI contains rich textural information, making it a promising candidate for improved measurement of lesion severity. Visually, the extent of T1 hypointense lesions reflects the persistence of tissue damage in MS (8). Quantitatively, measures of the “texture” of conventional MRI detect subtle structural abnormalities invisible to human eyes (9). Examples include characterization of brain white matter remodeling following traumatic brain injury (10), and separation of transient and persistent T1 hypointense lesions at onset in MS (11). In addition, based on texture analysis using a localized gray level co-occurrence matrix (GLCM) method and statistical machine learning using T2-weighted MRI of post-mortem brain samples, a prior study verified that texture metrics performed the best in classifying brain MS pathologies as compared to diffusion fractional anisotropy (FA) and magnetization transfer ratio (MTR) (12). Nonetheless, a criterion for identifying lesions of injury or repair in MS is still lacking in clinical imaging. Further, several demographic and clinical variables have shown the link to disease severity, such as older age and male sex to worse disease outcome (13, 14). Females are also evidenced to remyelinate more than males in MS (15, 16). However, the relationship between MRI-defined lesion severity and clinical variables is unclear.

The goal of this study was to identify the de- and re-myelination types of brain MS lesions in living participants using histology-verified MRI texture measures. The specific aims were to: (1) conduct whole brain MRI texture analysis in RRMS using an optimized GLCM technique; (2) classify the identified brain MS lesions in MRI into 2 extreme types of injury and repair including de- and re-myelination using a percentile ranking approach; and (3) evaluate differences between lesion types and their relationships with common demographic and clinical variables.

## 2. Materials and methods

### 2.1. Participants

This was a retrospective single-site study using data from a pilot clinical trial of domperidone as a candidate repair therapy in RRMS (clinicaltrials.gov; Identifier: NCT02493049). Recruitment occurred from November 2015 to January 2019, where 234 participants had complete screening. All of these individuals were under regular treatment with one of the approved disease modifying therapies (DMTs), and each of them had a clinically indicated brain MRI to screen for gadolinium-enhancing lesions for trial eligibility. After the screening brain MRI, eligible participants were randomized into one of the two treatment groups of the clinical trial, the details of which however were outside of the scope of this work. The current study was cross-sectional that focused only on the screening data acquired from a convenient sample of 200 participants (the first available individuals, one MRI per individual); 17 of them had at least one gadolinium-enhancing lesion on the screening brain MRI. This study was approved by the Institutional Ethics Board, and all participants provided written informed consent.

### 2.2. Imaging protocol

All MR images were acquired at a 3T scanner (Discovery750, GE Healthcare, Milwaukee, USA). The MRI protocol included clinical MRI involving T1-weighted and fluid-attenuated inversion recovery (FLAIR) images. Acquisition of T1-weighted MRI used a gradient recalled sequence, with the following parameters: repetition time (TR)/echo time (TE) = 8.1/3.1 ms, field of view (FOV) = 250 × 250 mm^2^, matrix = 256 × 256, and slice thickness = 1 mm. FLAIR MRI acquisition used a spin echo sequence, with TR/TE = 7,000/128 ms, FOV = 240 × 240 mm^2^, matrix = 512 × 512, and slice thickness = 1 mm. The imaging protocol also included other anatomical and advanced MRI sequences but were not the focus of the current study.

### 2.3. Image preparation

The MRI scans underwent several pre-processing steps to improve quality and uniformity. The process started with brain extraction, followed by linear co-registration from FLAIR to T1-weighted MRI, using the FSL software (Oxford, UK). The next step was noise reduction done using the ImageJ software (version 1.50i, NIH, USA), which involved median and mean filtering to remove salt and pepper and Gaussian noise, respectively, as commonly seen in MRI (17). The last step was signal intensity normalization to the range 0–255, using a customized coding program developed in-house.

### 2.4. Lesion segmentation

**Figure 1:**
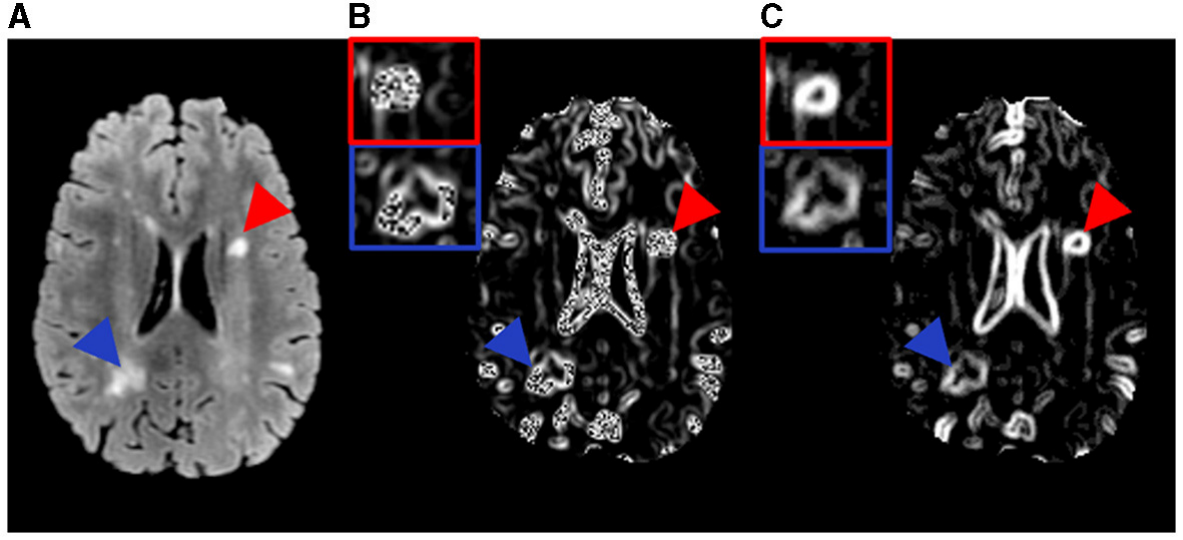
Example MR images and the texture feature maps from one RRMS participant examined in this study. Shown are a FLAIR image **(A)** as well as the resulting texture contrast **(B)** and texture dissimilarity **(C)** maps with corresponding lesions identified as severely demyelinated (red) or highly remyelinated (blue). Brighter signal indicates greater texture value and more heterogeneity in the maps. The participant has a disability score of 2.5 and disease duration of 17.1 years.

**Figure 2:**
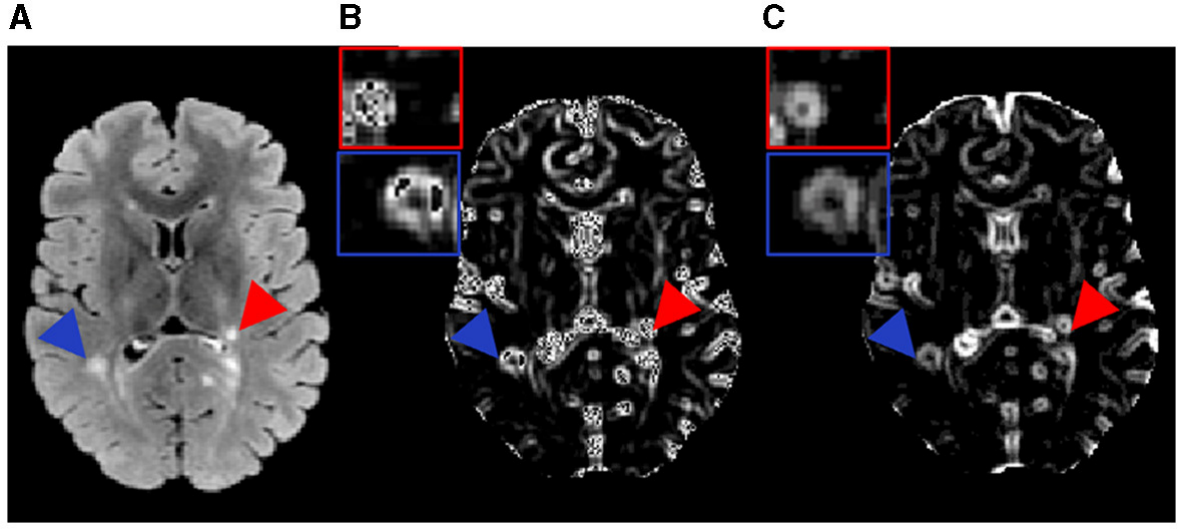
Example MR images and the texture feature maps from another RRMS participant examined in this study. Shown are a FLAIR image **(A)** as well as the resulting texture contrast **(B)** and texture dissimilarity **(C)** maps with corresponding lesions identified as severely demyelinated (red) or highly remyelinated (blue). Brighter signal indicates greater texture value and more heterogeneity in the maps. The participant has a disability score of 1.5 and disease duration of 9.5 years.

Focal lesions were identified using an automatic toolbox known as lesion segmentation tool (LST, v3.0.0) built in the software SPM12 (18). Lesion detection used the co-registered T1-weighted and FLAIR MRI scans. Subsequently, all lesion regions of interest (ROIs) were reviewed and manually corrected where applicable. In particular, lesions with pixels overlapping with the cerebral ventricles were adjusted to minimize partial volume effect or other undesired artifacts (19). For similar reasons, lesions with an area smaller than 5 pixels (~5 mm^2^) were excluded (Figures 1, 2).

### 2.5. Image texture analysis

Texture analysis employed the prepared FLAIR images where lesions were most distinguishable. Texture calculation used the GLCM method based on an optimized sliding-window approach. The GLCM evaluates the occurrence frequency of image pixels located in a certain distance and orientation relative to the neighboring pixels (20). Prior research showed that a distance of 1 (one) pixel was ideal for detecting fine image texture (21), and the average texture from all 4 common directions (0°, 45°, 90°, or 135°) of GLCM demonstrated the most promise in classifying lesion types (12, 22). Therefore, the above settings were used in the current study. The GLCM analysis focused on 2 measures: texture contrast and dissimilarity, which showed the best performance in classifying de- and re-myelinated lesions in brain white matter using histology-informed conventional MRI (12). Texture contrast was a measure of local variation in gray-level intensity, highlighting tissue coarseness. Texture dissimilarity computed the difference between gray-level pairs, indicating tissue heterogeneity. These measures were calculated from each sliding window sized 3 by 3. Iterating this process through the whole image provided the corresponding texture maps (Figures 1, 2). The GLCM analysis used the scikit-image library implementation in Python (version 2.7).

### 2.6. Lesion type definition

Classifying lesion types used a percentile thresholding approach. This was based on prior evidence suggesting that the coarseness of MRI texture was significantly greater in MS lesions with tissue damage than those with repair (11, 23). Further, to maximize understanding of texture differences, this study considered two lesion types expected to have the highest differences in tissue structure: severely demyelinated (sDEM), and highly remyelinated (hREM). Lesion texture maps from texture contrast and dissimilarity across all 200 participants were computed. Image texture map averaged from all four directions was derived per feature, and the mean texture value per lesion was used for percentile analysis in categorizing lesion types. Lesions with mean texture values ranked ≥75%^ile^ were considered sDEM, and ≤ 25%^ile^ as hREM.

### 2.7. Lesion type analysis

#### 2.7.1. Lesion size-based analysis

The average lesion size per type was measured firstly within a participant and then across participants. The calculation followed this equation: sum of mean lesion size per participant/total participants involved, where the mean lesion size in a participant was done by: sum of lesion area per type/number of lesions of the type in the participant. Additionally, the combined volume of each lesion type per subject was computed, which was normalized by the grand total lesion volume of the corresponding subject to account for lesion volume differences between participants. This total normalized volume of sDEM or hREM was used in statistical analyses.

#### 2.7.2. Lesion texture-based analysis

The mean texture value of each lesion type per subject was evaluated. In addition, based on the texture of individual lesions, principal component analysis (PCA) was performed to further explore the roles and relationships of the texture variables computed. This step took a correlation analysis approach. It indicated if the PCA variables (texture contrast and dissimilarity) were positively, negatively, or not correlated based on the location of the variables in a quadrant: grouped together, in opposing quadrants, or orthogonal to each other, respectively. Further, the contribution of each variable to the first and second PCs was calculated to evaluate how each measure reflected the degree of the underlying pathology.

#### 2.7.3. Lesion distribution mapping

**Figure 3:**
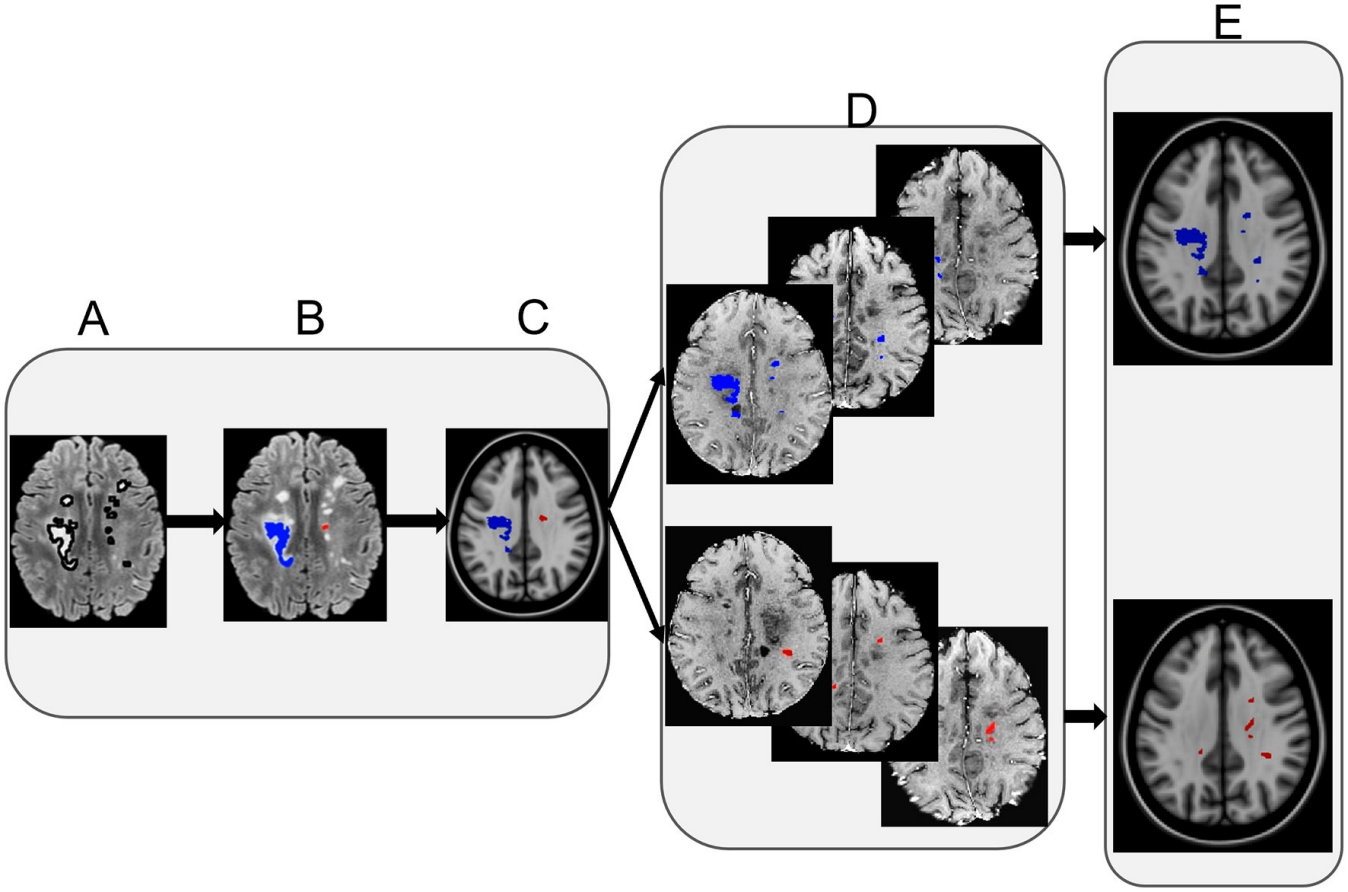
Lesion distribution map calculation. The left panel shows example FLAIR lesions segmented initially **(A)**, with lesion type identification **(B)**, and overlaid on a corresponding T1-weighted MRI co-registered to a common space [MNI152 1mm T1 template, **(C)**]. The middle panel shows example FLAIR lesions co-registered to the MNI template from individual participants per lesion type **(D)**. Finally, the right panel shows the eventual lesion masks averaged across participants following thresholding per lesion type **(E)**. Note: red represents the severely demyelinated lesions (sDEM) and blue refers to highly remyelinated lesions (hREM).

To understand the location characteristics of the identified lesion types in the brain, we generated lesion probability maps. Specifically, following brain extraction, the T1-weighted MRI of each subject was both linear (affine)- and non-linear- registered to the MNI152 1-mm T1-weighted MRI template as a common space (24). The resulting transformations from these co-registration steps were then applied with nearest neighbor interpolation to the respective T1-aligned lesion masks (Figure 3). In this way, the lesion masks from each subject were aligned pixel-wise with the MNI space. Averaging the masks across subjects for each lesion type generated the corresponding lesion probability maps. Finally, a threshold of 0.1 was applied to the masks such that lesions with a probability ≥10% was kept to improve reliability. In addition, lesion probability per type was explored by brain region, especially the white matter where MS lesions were most identifiable in brain MRI. The areas included: periventricular white matter, deep white matter, and corpus callosum. The corresponding area masks including lateral ventricles and brain white matter were extracted using established atlases in FSL. Subsequently, the mask of lateral ventricles were dilated with a disk size of 10 mm as indicated previously (25). Subtracting the lateral ventricle mask from its dilated version provided the periventricular white matter mask. The deep white matter mask was derived by subtracting the periventricular white matter mask from the eroded brain white matter mask with a kernel size of 1 mm (26). The overall distribution probability of lesions in an area per type was calculated as a ratio of the probability averaged across relevant image slices to the area volume for normalization purposes.

### 2.8. Relationship between lesion type and clinical variables

To explore the clinical relevance of the identified lesion types, we compared the average size and total normalized volume of each lesion type between sexes, and between younger and older groups both independently and within individual sex groups. The age groups were divided using a similar percentile approach. Participants with an age ≤ 25%^ile^ were classified as younger and ≥75%^ile^ as older. Further, the relationship between the aforementioned imaging measures per lesion type and other clinical measures including disease duration, expanded disability status scale (EDSS) score at screening, use of DMTs including high vs. moderate efficacy DMTs were also assessed.

### 2.9. Statistical analysis

All statistical analyses used R (version 3.6.3) (27). Data normality assessments employed the Shapiro-Wilk test. Outcome comparisons between two groups used the Wilcoxon signed-rank test, including comparisons between lesion types and between clinical variables. Comparison on the probability of lesion distribution between different brain areas used the non-parametric Kruskal-Wallis test. Further, relationship assessment between imaging and clinical measures applied the Pearson correlation for continuous variables, and Spearman Correlation for categorical variables such as EDSS. In all analyses, a *p* < 0.05 was considered significant.

## 3. Results

### 3.1. Sample characteristics

There were 5,140 lesions evaluated in total from the 200 RRMS participants (148 females). Lesion size ranged 5–342 mm^2^. Of all the participants, the EDSS ranged 0–6.5, and disease duration ranged 1.4–35.6 years. Participant age ranged 20.4 to 60.3 years, and it was 20.4–60.3 years for women and 21.0–60.0 years for men (Table 1). All participants were under regular clinical management including treatment with up to ten different DMTs, such as dimethyl fumarate, fingolimod, glatiramer acetate, interferon-beta, and teriflunomide as the most common ones.

**Table 1:** Demographic characteristics of the RRMS participants at screening.

**Variable**	**Mean (range)**	**SD**
Age (years)	44.4 (20.4–60.3)	8.8
Sex (female/male)	148/52	–
Lesion volume (mm^3^)	2290 (13.4–24727.0)	3216.0
Lesion number all types	25.50 (1–190)	24.60
sDEM lesions	27.82 (0–59)	31.21
hREM lesions	26.92 (0–66)	28.71
EDSS	2.25 (0.0–6.5)	1.51
Disease duration	11.75 (1.40–35.60)	7.28

### 3.2. Lesion type outcomes

Based on our percentile-thresholding approach, 193 of 200 RRMS participants (96.5%) had both sDEM and hREM detected; 143 were women. Four of the 200 participants had only sDEM lesions (all women), and three had only hREM lesions (one woman).

#### 3.2.1. The sDEM had a smaller average size but larger total normalized volume than hREM

**Figure 4:**
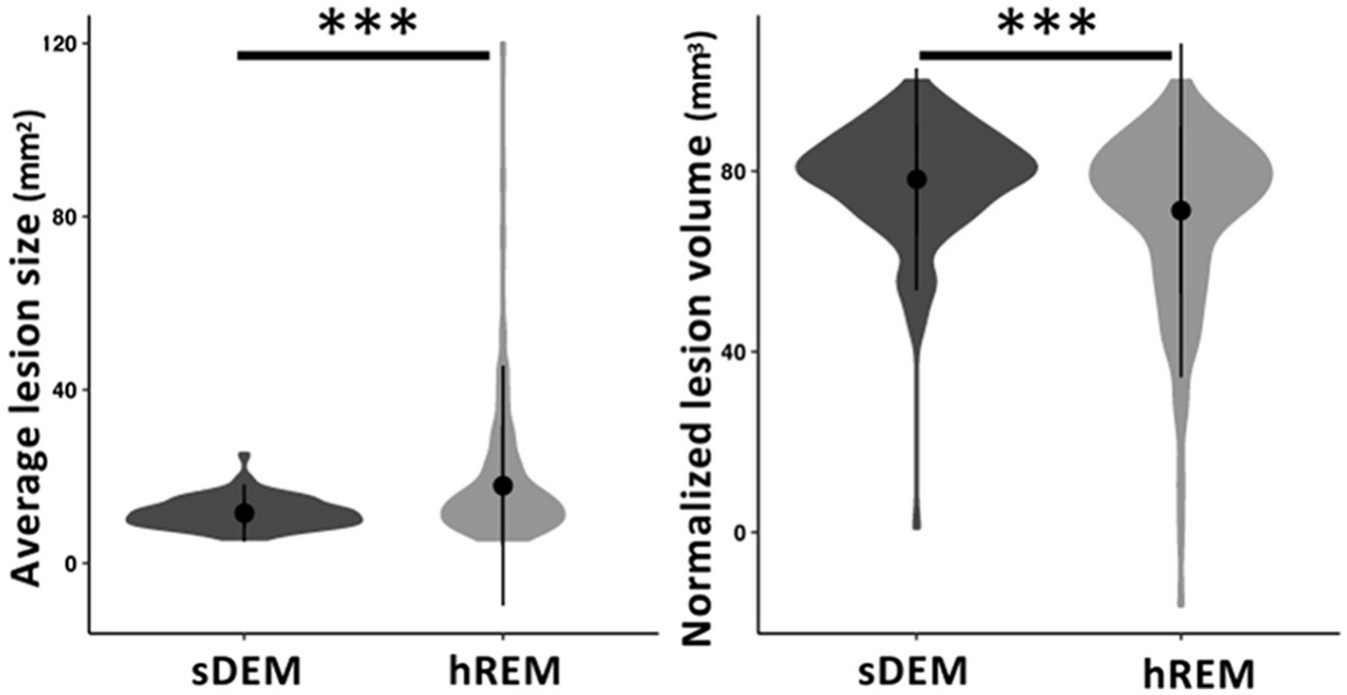
Average size and normalized volume of the two identified lesion types across all RRMS participants. Shown are violin plots of the mean, standard deviation, and range (density) of the lesion values. sDEM, severely demyelinated lesions; hREM, highly remyelinated lesions. ^***^p < 0.001.

The average lesion size differed between participants in each lesion type. It ranged 5.72–25.11 mm^2^ for sDEM and 5.4–119.8 mm^2^ for hREM across the corresponding cohorts. Wilcoxon signed-rank test revealed that the sDEM possessed a significantly smaller average size than the hREM (350.42 mm^2^ vs. 660.17 mm^2^, *p* < 0.001). Conversely, the total normalized lesion volume was significantly larger in sDEM than hREM (78.24% vs. 71.34%, *p* < 0.001) (Figure 4).

#### 3.2.2. Texture contrast and dissimilarity contributed similarly in classifying sDEM and hREM

**Figure 5:**
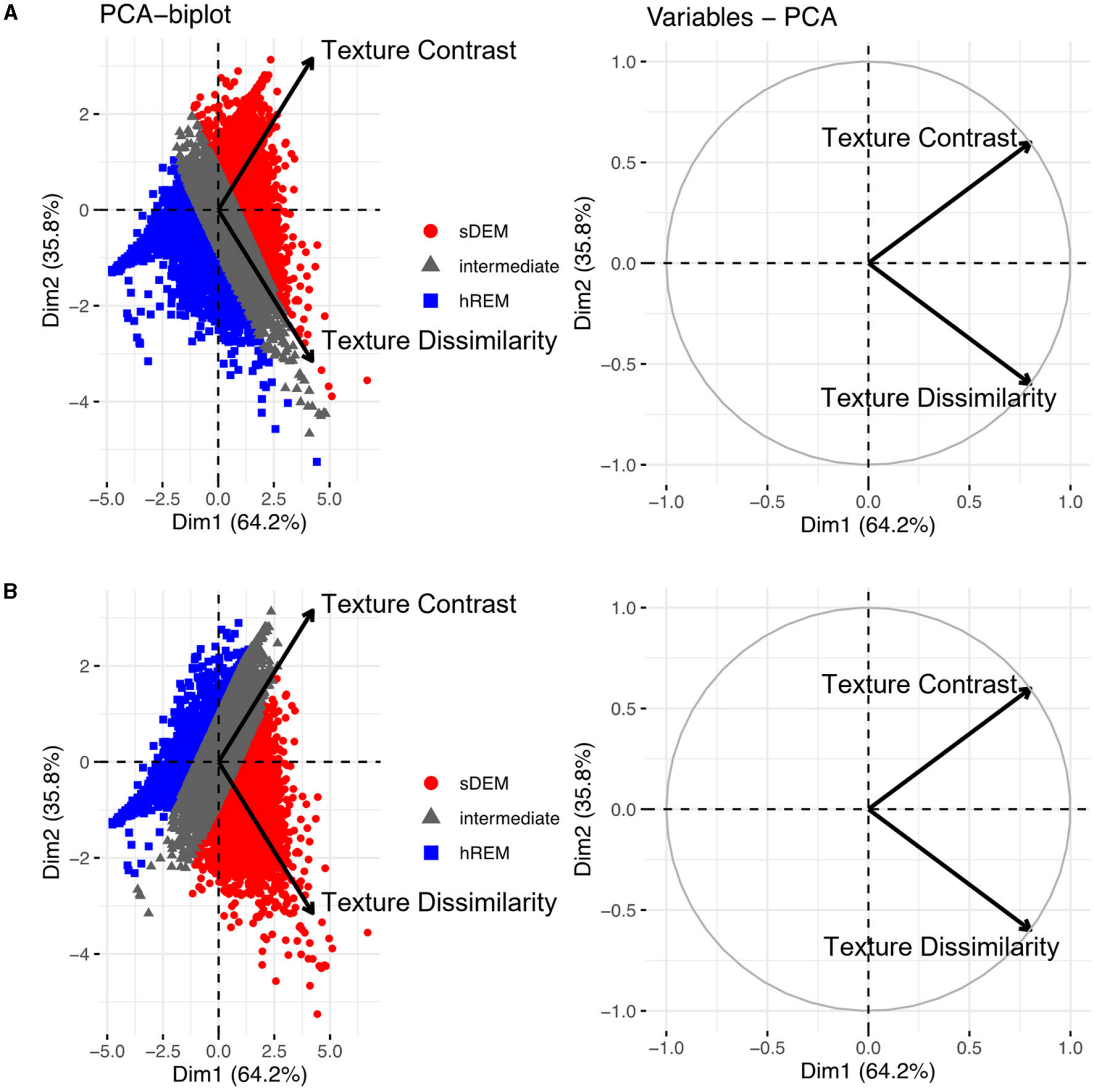
Principal component analysis (PCA) outcomes. The PCA biplots (left) show clusters of lesion texture values based on their similarity and the strength of individual features influencing a principal component. Shown are the 2 types of lesions identified in this study based on texture contrast **(A)** or texture dissimilarity **(B)**, and the remaining lesions with intermediate texture values (gray). The PCA variable plots (right) show the relationship and contribution of the features. sDEM, severely demyelinated lesions; hREM, highly remyelinated lesions.

The mean (standard deviation) texture contrast for sDEM and hREM was 38.59 (1.42) and 21.66 (2.27), respectively. Similarly, texture dissimilarity was 9.86 (1.40) and 3.82 (0.37) for sDEM and hREM. The PCA analysis of texture variables from individual lesions showed that the sDEM was clearly separable from the hREM based on either texture contrast or dissimilarity. The first PC explained 64.2% of the variance, whereas the second PC carried 35.8% of the variation. Further, the two texture variables appeared almost orthogonal and therefore they had a weak or no correlation with each other. Moreover, the contribution of the 2 texture variables to the PCs was ~50% each (Figure 5).

#### 3.2.3. Probability distribution of the identified lesion types

**Figure 6:**
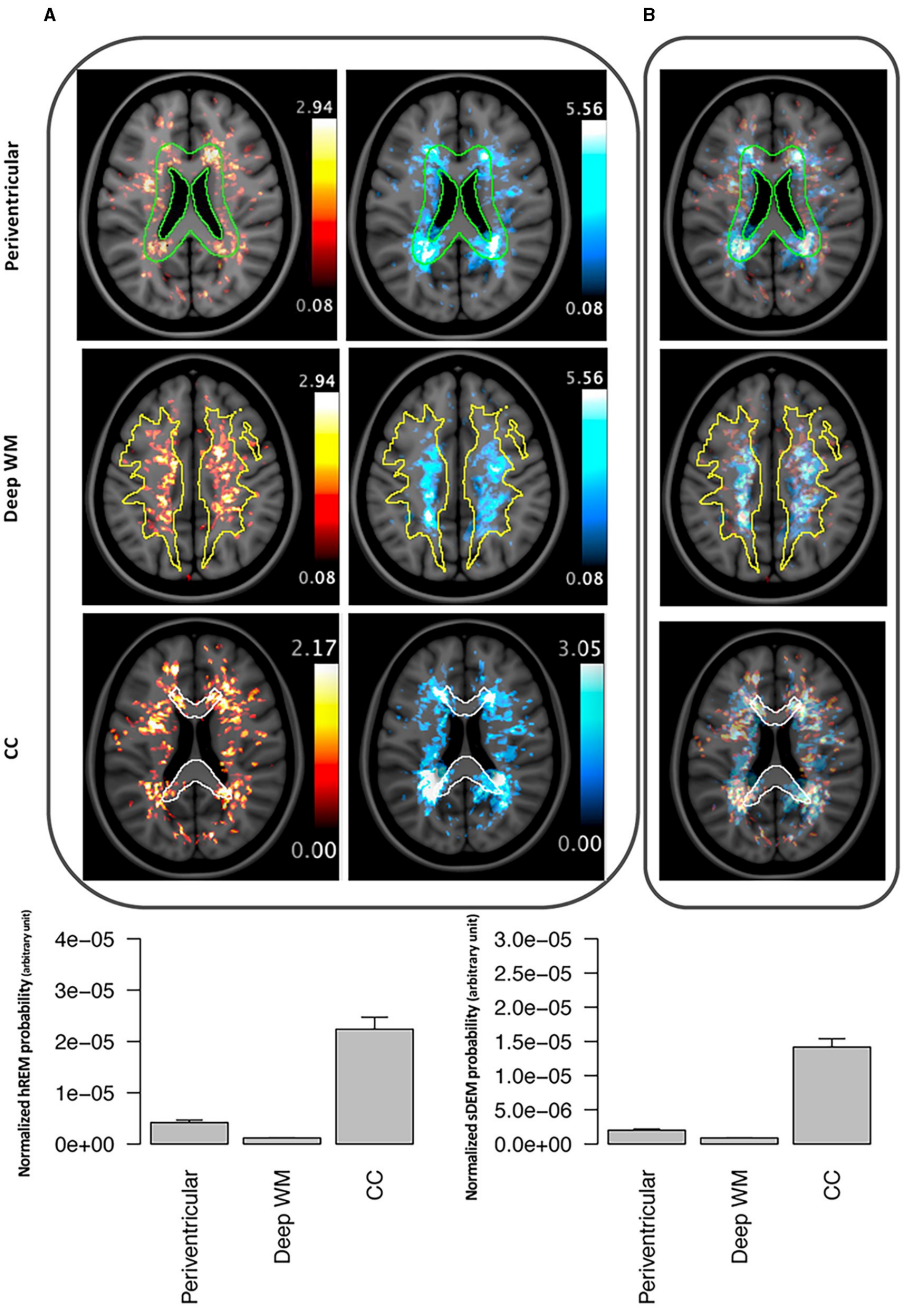
Example lesion distribution maps and area-wise quantification across all RRMS participants. Top panel shows the distribution of severely demyelinated (red) and highly remyelinated (blue) lesions separately **(A)** and together **(B)** at 3 commonly recognized brain areas in MS: periventricular, deep white matter (WM), and corpus callosum (CC). All images represent corresponding MRI slices from the co-registered MNI template. The outlines wherein represent the area masks generated from the template. Bottom panel shows the mean (standard error) probability of each lesion type within each of the 3 defined brain areas. Individual probabilities are normalized by the volume of the corresponding anatomical area. sDEM, severely demyelinated lesions; hREM, highly remyelinated lesions.

Both lesion types showed a high probability of presence in the cerebral white matter along with substantial overlap in location. Quantitatively, the mean (standard deviation) probability of distribution was the highest in the corpus callosum, which was 50% (41%) for sDEM and 79% (78%) for hREM. This was followed by the periventricular white matter at 29% (24%) and 60% (69%), and deep white matter at 22% (16%) and 30% (26%) for sDEM and hREM, respectively. On average, up to 1% of the lesions from both types was distributed in the cerebellum and brainstem areas, and 2% of the lesions were seen in deep gray matter. Further, the hREM showed a much more concentrated distribution than the sDEM, seen mainly around the lateral ventricles, corpus callosum, and deep white matter (Figure 6).

### 3.3. Lesion type outcomes in relation to clinical measures

**Figure 7:**
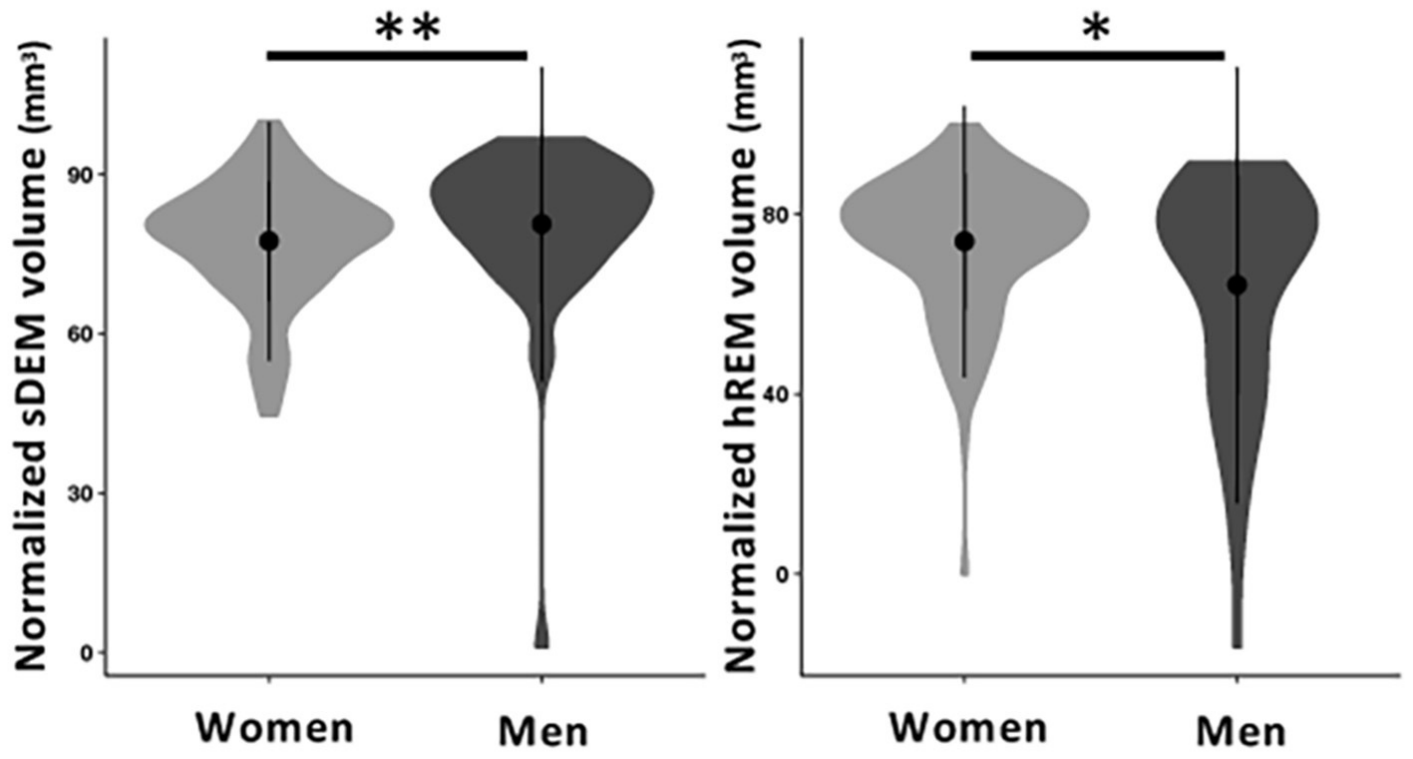
Normalized volume of sDEM and hREM in women and men RRMS participants. Shown are violin plots of the mean, standard deviation, and range (density) of lesion texture values. sDEM, severely demyelinated lesions; hREM, highly remyelinated lesions. ^*^p < 0.05; ^**^p < 0.01.

Between sexes, men had a significantly higher total normalized volume of sDEM than women [mean (standard deviation) = 80.61 (14.78) mm^3^ vs. 77.41 (11.22) mm^3^ *p* = 0.01]. Conversely, women had a higher total normalized volume of hREM lesions than men [73.93 (15.11) mm^3^ vs. 64.26 (24.24) mm^3^, *p* = 0.02; Figure 7]. The average lesion size was not significantly different between women and men in sDEM [11.3 (3.3) mm^2^ in women vs. 12.1 (3.1) mm^2^ in men, *p* = 0.13] or hREM [21.21 (17.84) mm^2^ vs. 16.64 (11.83), *p* = 0.06]. Further, there were no significant differences between younger and older groups in either average lesion size or total normalized volume of sDEM or hREM, when assessed independently or within individual sex groups. In correlation analyses, there was no significant relationship between the average lesion size or total normalized lesion volume and disease duration, EDSS, and DMT use or efficacy.

## 4. Discussion

In this study, we developed a percentile thresholding approach to identify 2 critical types of brain MS lesions based on histology-informed texture measures of conventional MRI. Using recognized percentile thresholds, this study found that most of the RRMS participants had both sDEM and hREM. In comparison, the total normalized volume of sDEM were greater than hREM, but the average size of hREM was larger than sDEM. Based on the PCA, texture contrast and dissimilarity were uncorrelated and contributed similarly in lesion separation. Further, both types of lesions showed a high likelihood of distribution in the corpus callosum, and then periventricular and deep white matter, with the hREM appearing more aggregated than sDEM. Furthermore, women had less sDEM but greater hREM in total normalized volume than men; instead, men showed a trend for a larger size in hREM than women.

Texture is an intrinsic characteristic of a tissue structure and can be determined by the degree of coarseness, fineness, irregularity, and complexity. Image textural information has shown to be valuable for tissue discrimination and classification (20). In general, a repaired tissue is expected to exhibit a fine imaging texture, whereas a damaged tissue such as demyelination would create a heterogeneous texture pattern (9, 28). The GLCM contrast and dissimilarity are leading measures of texture coarseness and heterogeneity (20, 29). Indeed, we detected greater texture contrast and dissimilarity in sDEM than hREM in the present study. Our PCA analyses further detected that the two GLCM texture features were independent and contributed similarly to the major PCs in lesion type identification based on individual ROI values, suggesting that using either texture feature alone may be feasible in similar studies.

With respect to lesion type analyses, this study detected a smaller average lesion size but greater total normalized lesion volume in sDEM than hREM. One of the critical reasons for sustained disease progression in MS is the lack of remyelination or repair (30, 31). Therefore, it was not surprising to find in the present study that the total normalized lesion volume of sDEM was significantly greater than hREM. Previously, based on diffusion MRI measures, a study performed cluster analysis that grouped MS lesions into 2 severity types. They found that the volume of more severe lesions was higher than less severe lesions (4). Regarding lesion size, larger lesions would be expected to have more heterogeneous structure due to the higher likelihood of possessing inhomogeneous tissue pathology across the lesion area. On the other hand, patches of remyelination might also more likely be present in larger lesions, given the evidence of preferably uneven repair across lesion regions in MS lesions as documented in histology (32, 33). Additionally, the sDEM could also have had a high degree of tissue loss, causing an atrophic change and therefore reduced size, which in turn limited their capacity for repair such as remyelination. Combined results might have contributed to the relatively worse MRI texture regularity in sDEM than hREM. Nonetheless, a prior study based on postmortem brains of two progressive MS participants had also reported that smaller lesions might remyelinate more effectively than larger ones (34). But the sample size therein was small and disease phenotypes were different, deserving further investigation.

Our implementation of lesion distribution maps served as another valuable means to understand the identified lesion types. Previously, several studies have used lesion distribution maps to investigate lesion development in MS. One of them compared the patterns of lesion distribution between RRMS and SPMS (35). They showed that the periventricular area was more subject to severe tissue injury than other brain white matter regions and the damage was more pronounced in SPMS than RRMS. Another study analyzed the change in spatiotemporal patterns of active lesions over time in RRMS, where they found that there was a reduction of active lesion development in major white matter tracts such as corticospinal tract after treatment (36). Nevertheless, there was lack of information on the distribution of injury and repair lesions in MS. In the current study, our probability distribution maps highlighted that both sDEM and hREM were highly distributed in the corpus callosum, followed by the periventricular and deep white matter regions. While previous studies indicated that remyelination occurred more frequently in the subcortical than periventricular brain white matter in MS (37, 38), our findings suggest that strong remyelination repair may also happen in other brain areas. Alternatively, our results may not be completely contrary to prior evidence because our hREM only represent an extreme group of lesions that likely had the most degree of remyelination, not lesions with intermediate or mild degrees of repair. In addition, our associated analyses in this study also demonstrated the scarcity of brainstem lesions from both types. This might be due to several factors, including the inherent lack of occurrence of MS lesions in the brainstem, and the challenge of imaging lesions in this region (39). Furthermore, brainstem lesions are subject to early apoptotic changes in oligodendrocytes (40) and it is not uncommon for brainstem to have significant volume loss in RRMS (41).

This study detected significant differences in total normalized lesion volume between sexes but no other significant results were found in terms of other demographic and clinical variables. Besides the strong differences in disease prevalence, women and men also seem to show distinct disease outcomes in MS. Once initiated, the disease is likely to worsen faster in men than women (42, 43). While not fully understood, the higher total normalized volume in sDEM but lower in hREM found in men in our study may indicate reduced repair extent as compared to women. Evidence on the impact of age on MS disease activity is mixed in the literature. Various studies have reported that the repair potential decreases in older MS participants than younger ones (44, 45). However, this result was not supported by a recent study (46). Based on 30 RRMS participants, the authors investigated the effect of age on intralesional tissue evolution using different MRI measures including neurite density and orientation dispersion indices, MTR, and T1 relaxometry. By dividing participants into young (age <25^th^ percentile) and old (age > 75^th^ percentile) groups as done in the current study, they discovered that age did not affect the repair pattern of MS lesions in MRI. In addition, we did not find significant relationships between identified lesion types and the investigated clinical measures here, and associated evidence in the literature is scarce. One of the few positive studies showed that based on diffusion MRI measurements, the number and volume of relatively severe MS lesions were associated with disease severity and cognitive decline (4). But the participant characteristics, sample size, and MR metrics used were different between prior and current studies, and therefore direct comparison of results is difficult.

Percentile statistics has shown enormous promise in classifying tissue types or disease activity in various studies, including those in MS (6, 47, 48). Based on MTR signal intensity inhomogeneity, a previous study used the same 25%^ile^ and 75%^ile^ thresholds to define tissues of highly repairing and highly damaging potential in MS lesions and normal appearing white matter (49). In another study, by comparing different thresholds, the authors showed that the 25%^ile^ and 75%^ile^ thresholds of texture contrast and dissimilarity were most feasible in differentiating de-and re-myelinated MS lesions using histology-verified T2-weighted MRI (50). Similarly, the percentile approach has also shown the utility in differentiating disease activities in MS. One study divided relapsing and progressive MS by using lesion and brain volume, and lesional myelin water fraction (MWF), and they discovered that the median, 25%^ile^, and 75%^ile^ of MWF, and 75%^ile^ of lesion volume were the top ranking features (51). Another study differentiated the severity of RRMS participants based on their 25%^ile^ and 75%^ile^ of lesion load, where the 2 groups showed significant differences in brain microstructure as measures by diffusion MRI (48). In the present study, we adopted the 25%^ile^ and 75%^ile^ thresholds based on two top-performing GLCM features (contrast and dissimilarity) as demonstrated in a prior MRI texture-histology study (12). Our observation that most of the RRMS participants showed both sDEM and hREM is in accordance with recent evidence showing that both lesion types exist in most MS subjects, with only some individuals presenting with a dominant lesion type (4).

Our study has several limitations. The focus was mainly on cerebral white matter lesions. While lesion injury and repair may be present in other parts of the brain including gray matter, the main purpose of this study was to investigate whether and how a method for lesion type characterization can be derived. Given the prevalence of inflammatory changes across the brain in RRMS and the sensitivity of conventional MRI to these changes in brain white matter, studying white matter pathology seems reasonable. In addition, as part of a wellrecognized challenge in imaging of living people in MS, it was unlikely to differentiate lesions fallen between the two defined thresholds (75%^ile^ and 25%^ile^) in this study, such as lesions of incomplete demyelination vs. partial remyelination. In a similar sense, the hREM type might also have included lesions of mild demyelination although both mild injury and heavy repair would similarly represent a positive benefit. The aforementioned lesion differentiation challenges were likely exacerbated by the lack of longitudinal data or direct histological analysis for confirmation that formed other limitations of the study. However, this study aimed to characterize lesions of “extreme” injury or repair that was expected to be highly reflective of de- and re-myelination. This expectation could be attributed to several factors, including the commonly demyelinating nature of MS pathology (2), the likelihood of lesion repair with remyelination rather than axonal regrowth (52, 53), and the relative utility of the texture measures for assessing de- and re-myelinated brain MS lesions as shown previously (12). Therefore, based on recognized percentile thresholds, our identification of such extreme types of lesions appeared reasonable. Further, this study was limited to RRMS. Combining other MS subtypes may broaden the scope of the study. However, RRMS is the phenotype that has the most lesions with active de- and re-myelination. Finally, this study focused on lesion types that represented approximately half instead of all of the lesion counts from the whole sample size involved. At an individual level, the sDEM and hREM in combination may represent most or minimal amount of lesions in a participant depending on disease activity or severity. While with limitations, this approach allowed to assess lesions of potentially the most influence and obtain results with the highest confidence. Obtaining these results *in vivo* using routinely available brain MRI scans would further help promote the power of clinical imaging, in addition to adding likely new information to the literature. In the future, we seek to verify our findings using different datasets, assess other types of lesions in different locations, and correlate MRI results with different types of measures of disease development or intervention impact.

In summary, characterization of lesion severity *in vivo* is fundamental for a thorough understanding of disease activity and treatment impact in MS. Using histology-verified brain MRI texture measures and a simple percentile thresholding approach, this study shows the potential to identify two critical types of brain MS lesions. With further confirmation, this information can help improve our ability in disease monitoring and in identifying new reparative therapies in MS. Further, the different outcomes of the identified lesions between men and women may stimulate new sex-specific studies, including their relationship with clinical outcomes and the development of associated intervention and prevention strategies to improve the prognosis of all individuals with MS.

## Data availability statement

The original contributions presented in the study are included in the article/supplementary material, further inquiries can be directed to the corresponding author.

## Ethics statement

The studies involving human participants were reviewed and approved by Conjoint Health Research Ethics Board. The patients/participants provided their written informed consent to participate in this study.

## Author contributions

ZH: conceptualization, methodology, data curation, formal analysis, writing—original draft, review, and editing. OO: methodology. W-qL: resources. GP and VY: writing—review and editing and supervision. LM: writing—review and editing. YZ: conceptualization, writing—review and editing, resources, supervision, and funding acquisition. All authors contributed to the article and approved the submitted version.

## References

[1] Lassiter G Melancon C Rooney T Murat A-M Kaye JS Kaye AM. Ozanimod to treat relapsing forms of multiple sclerosis: a comprehensive review of disease, drug efficacy and side effects. Neurol Int. (2020) 12:89–108. 10.3390/neurolint12030016 33287177 PMC7768354

[2] Lassmann H. Multiple sclerosis pathology. Cold Spring Harb Perspect Med. (2018) 8:a028936. 10.1101/cshperspect.a028936 29358320 PMC5830904

[3] Lipp I Jones DK Bells S Sgarlata E Foster C Stickland R. Comparing MRI metrics to quantify white matter microstructural damage in multiple sclerosis. Hum Brain Mapp. (2019) 40:2917–32. 10.1002/hbm.24568 30891838 PMC6563497

[4] Martínez-Heras E Solana E Prados F Andorrà M Solanes A López-Soley E. Characterization of multiple sclerosis lesions with distinct clinical correlates through quantitative diffusion MRI. NeuroImage Clin. (2020) 28:102411. 10.1016/j.nicl.2020.102411 32950904 PMC7502564

[5] Klauser AM Wiebenga OT Eijlers AJ Schoonheim MM Uitdehaag BM Barkhof F. Metabolites predict lesion formation and severity in relapsing-remitting multiple sclerosis. Multiple Sclerosis J. (2018) 24:491–500. 10.1177/1352458517702534 28406063

[6] Reich DS White R Cortese IC Vuolo L Shea CD Collins TL. Sample-size calculations for short-term proof-of-concept studies of tissue protection and repair in multiple sclerosis lesions via conventional clinical imaging. Multiple Sclerosis J. (2015) 21:1693–704. 10.1177/1352458515569098 25662351 PMC4527958

[7] Xiang B Wen J Cross AH Yablonskiy DA. Single scan quantitative gradient recalled echo MRI for evaluation of tissue damage in lesions and normal appearing gray and white matter in multiple sclerosis. J Magn Reson Imag. (2019) 49:487–98. 10.1002/jmri.26218 30155934 PMC6423972

[8] Bagnato F Jeffries N Richert ND Stone RD Ohayon JM Mcfarland HF. Evolution of T1 black holes in patients with multiple sclerosis imaged monthly for 4 years. Brain. (2003) 126:1782–9. 10.1093/brain/awg182 12821527

[9] Zhang Y. MRI texture analysis in multiple sclerosis. Int J Biomed Imaging. (2012) 12:762804. 10.1155/2012/762804 22144983 PMC3227516

[10] Fozouni N Chopp M Nejad-Davarani SP Zhang ZG Lehman NL Gu S. Characterizing brain structures and remodeling after TBI based on information content, diffusion entropy. PLoS One. (2013) 8:e76343. 10.1371/journal.pone.0076343 24143186 PMC3797055

[11] Zhang Y Traboulsee A Zhao Y Metz LM Li DK. Texture analysis differentiates persistent and transient T1 black holes at acute onset in multiple sclerosis: a preliminary study. Multiple Sclerosis J. (2011) 17:532–40. 10.1177/1352458510395981 21270058

[12] Hosseinpour Z Jonkman L Oladosu O Pridham G Pike GB Inglese M. Texture analysis in brain T2 and diffusion MRI differentiates histology-verified grey and white matter pathology types in multiple sclerosis. J Neurosci Methods. (2022) 109671. 10.1016/j.jneumeth.2022.109671 35820450

[13] Harbo HF Gold R Tintoré M. Sex and gender issues in multiple sclerosis. Therapeutic Adv Neurologic Disord. (2013) 6:237–48. 10.1177/1756285613488434 23858327 PMC3707353

[14] Bove R Chitnis T. The role of gender and sex hormones in determining the onset and outcome of multiple sclerosis. Multiple Sclerosis J. (2014) 20:520–6. 10.1177/1352458513519181 24561324

[15] Dunn SE Gunde E Lee H. Sex-based differences in multiple sclerosis (MS): part II: rising incidence of multiple sclerosis in women and the vulnerability of men to progression of this disease. Emerg Evolv Topics Multiple Sclerosis Pathogen Treat. (2015) 15:57–86. 10.1007/7854_2015_370 25690592

[16] Li W-W Penderis J Zhao C Schumacher M Franklin RJ. Females remyelinate more efficiently than males following demyelination in the aged but not young adult CNS. Exp Neurol. (2006) 202:250–4. 10.1016/j.expneurol.2006.05.012 16797535

[17] Isa IS Sulaiman SN Mustapha M Darus S. Evaluating denoising performances of fundamental filters for t2-weighted MRI images. Procedia Comput Sci. (2015) 60:760–8. 10.1016/j.procs.2015.08.231

[18] Schmidt P Gaser C Arsic M Buck D Förschler A Berthele A. An automated tool for detection of FLAIR-hyperintense white-matter lesions in multiple sclerosis. Neuroimage. (2012) 59:3774–83. 10.1016/j.neuroimage.2011.11.032 22119648

[19] Herlihy AH Hajnal JV Curati WL Virji N Oatridge A Puri BK. Reduction of CSF and blood flow artifacts on FLAIR images of the brain with k-space reordered by inversion time at each slice position (KRISP). Am J Neuroradiol. (2001) 22:896–904. 11337335 PMC8174936

[20] Haralick RM Shanmugam K Dinstein IH. Textural features for image classification. IEEE Transact Systems Man Cybernetics. (1973) 12:610–621. 10.1109/TSMC.1973.4309314

[21] Gebejes A Huertas R. Texture characterization based on grey-level co-occurrence matrix. Databases. (2013) 9:258.

[22] Clausi DA. An analysis of co-occurrence texture statistics as a function of grey level quantization. Canad J Rem Sens. (2002) 28:45–62. 10.5589/m02-00422759441

[23] Zhang Y Zhu H Mitchell JR Costello F Metz LM. T2 MRI texture analysis is a sensitive measure of tissue injury and recovery resulting from acute inflammatory lesions in multiple sclerosis. Neuroimage. (2009) 47:107–11. 10.1016/j.neuroimage.2009.03.075 19361563

[24] Grabner G Janke AL Budge MM Smith D Pruessner J Collins DL. Symmetric atlasing and model based segmentation: an application to the hippocampus in older adults. Int Conf Med Image Comput Comput Assist Intervent. (2006) 6:58–66. 10.1007/11866763_8 17354756

[25] Griffanti L Jenkinson M Suri S Zsoldos E Mahmood A Filippini N. Classification and characterization of periventricular and deep white matter hyperintensities on MRI: a study in older adults. Neuroimage. (2018) 170:174–81. 10.1016/j.neuroimage.2017.03.024 28315460

[26] Park B-Y Lee MJ Lee S-H Cha J Chung C-S Kim ST. DEWS (DEep White matter hyperintensity Segmentation framework): a fully automated pipeline for detecting small deep white matter hyperintensities in migraineurs. NeuroImage: Clinic. (2018) 18:638–47. 10.1016/j.nicl.2018.02.033 29845012 PMC5964963

[27] R Core Team. R: A language and environment for statistical computing. R Foundation for Statistical Computing (2020).

[28] Zhang Y Jonkman L Klauser A Barkhof F Yong VW Metz LM. Multi-scale MRI spectrum detects differences in myelin integrity between MS lesion types. Multiple Sclerosis J. (2016) 22:1569–77. 10.1177/1352458515624771 26754802

[29] Larue RT Defraene G De Ruysscher D Lambin P Van Elmpt W. Quantitative radiomics studies for tissue characterization: a review of technology and methodological procedures. Br J Radiol. (2017) 90:20160665. 10.1259/bjr.20160665 27936886 PMC5685111

[30] Correale J Ysrraelit MC. Multiple Sclerosis and Aging: The Dynamics of Demyelination and Remyelination. ASN Neuro. (2022) 14:17590914221118502. 10.1177/17590914221118502 35938615 PMC9364177

[31] Cunniffe N Coles A. Promoting remyelination in multiple sclerosis. J Neurol. (2021) 268:30–44. 10.1007/s00415-019-09421-x 31190170 PMC7815564

[32] Macchi M Magalon K Zimmer C Peeva E El Waly B Brousse B. Mature oligodendrocytes bordering lesions limit demyelination and favor myelin repair via heparan sulfate production. Elife. (2020) 9:e51735. 10.7554/eLife.51735.sa2 32515730 PMC7308090

[33] Kuhlmann T Ludwin S Prat A Antel J Brück W Lassmann H. An updated histological classification system for multiple sclerosis lesions. Acta Neuropathol. (2017) 133:13–24. 10.1007/s00401-016-1653-y 27988845

[34] Patani R Balaratnam M Vora A Reynolds R. Remyelination can be extensive in multiple sclerosis despite a long disease course. Neuropathol Appl Neurobiol. (2007) 33:277–87. 10.1111/j.1365-2990.2007.00805.x 17442065

[35] Filli L Hofstetter L Kuster P Traud S Mueller-Lenke N Naegelin Y. Spatiotemporal distribution of white matter lesions in relapsing–remitting and secondary progressive multiple sclerosis. Multiple Sclerosis J. (2012) 18:1577–84. 10.1177/1352458512442756 22495945

[36] Giorgio A Battaglini M Gentile G Stromillo ML Gasperini C Visconti A. Mapping the progressive treatment-related reduction of active MRI lesions in multiple sclerosis. Front Neurol. (2020) 20:1466. 10.3389/fneur.2020.585296 33329329 PMC7714945

[37] Patrikios P Stadelmann C Kutzelnigg A Rauschka H Schmidbauer M Laursen H. Remyelination is extensive in a subset of multiple sclerosis patients. Brain. (2006) 129:3165–72. 10.1093/brain/awl.217 16921173

[38] Goldschmidt T Antel J König F Brück W Kuhlmann T. Remyelination capacity of the MS brain decreases with disease chronicity. Neurology. (2009) 72:1914–21. 10.1212/WNL.0b013e3181a8260a 19487649

[39] Habek M. Evaluation of brainstem involvement in multiple sclerosis. Expert Rev Neurother. (2013) 13:299–311. 10.1586/ern.13.18 23448219

[40] Ludwin SK. The pathogenesis of multiple sclerosis: relating human pathology to experimental studies. J Neuropathol Exp Neurol. (2006) 65:305–18. 10.1097/01.jnen.0000225024.12074.80 16691112

[41] Elzayady M Debees NL Khalil M Dawoud MM. Cerebellum and brain stem volume loss in relapsing remission multiple sclerosis by MRI volumetry: relation to neurological disability score and number of relapses. Egyptian J Radiol Nucl Med. (2021) 52:1–9. 10.1186/s43055-020-00394-w

[42] Avila M Bansal A Culberson J Peiris AN. The role of sex hormones in multiple sclerosis. Eur Neurol. (2018) 80:93–9. 10.1159/000494262 30343306

[43] Schwendimann RN Alekseeva N. Gender issues in multiple sclerosis. Int Rev Neurobiol. (2007) 79:377–92. 10.1016/S0074-7742(07)79017-7 17531851

[44] Neumann B Segel M Chalut KJ Franklin RJ. Remyelination and ageing: Reversing the ravages of time. Multiple Sclerosis J. (2019) 25:1835–41. 10.1177/1352458519884006 31687878 PMC7682531

[45] Rist JM Franklin RJ. Taking ageing into account in remyelination-based therapies for multiple sclerosis. J Neurol Sci. (2008) 274:64–7. 10.1016/j.jns.2008.04.027 18539300

[46] Fischi-Gomez EFJ Mario B Guillaume GC. The age effect on multi-parametric magnetic resonance imaging changes in multiple sclerosis lesions. In: Proceedings of the International Society for Magnetic Resonance in Medicine. (2018). p. 183.

[47] Chen JT Collins DL Atkins HL Freedman MS Arnold DL Group CMBS. Magnetization transfer ratio evolution with demyelination and remyelination in multiple sclerosis lesions. Ann Neurol. (2008) 63:254–62. 10.1002/ana.21302 18257039

[48] Oladosu O Liu W-Q Pike BG Koch M Metz LM Zhang Y. Advanced analysis of diffusion tensor imaging along with machine learning provides new sensitive measures of tissue pathology and intra-lesion activity in multiple sclerosis. Front Neurosci. (2021) 15:540. 10.3389/fnins.2021.634063 34025338 PMC8138061

[49] Chen J Collins DL Freedman M Atkins H Arnold DL Group CMBS. Local magnetization transfer ratio signal inhomogeneity is related to subsequent change in MTR in lesions and normal-appearing white-matter of multiple sclerosis patients. Neuroimage. (2005) 25:1272–8. 10.1016/j.neuroimage.2004.12.046 15850745

[50] Hosseinpour Z Oladosu O Soleymani M Pike BG Zhang Y. Characterization of multiple sclerosis lesion types with texture analysis of advanced and conventional MRI. In: Proceedings of the International Society for Magnetic Resonance in Medicine. (2021). p. 258.35820450

[51] Hurtado Rúa SM Kaunzner UW Pandya S Sweeney E Tozlu C Kuceyeski A. Lesion features on magnetic resonance imaging discriminate multiple sclerosis patients. European J Neurol. (2022) 29:237–46. 10.1111/ene.15067 34402140 PMC8727028

[52] Franklin RJ Ffrench-Constant C Edgar JM Smith KJ. Neuroprotection and repair in multiple sclerosis. Nat Rev Neurol. (2012) 8:624–34. 10.1038/nrneurol.2012.200 23026979

[53] Rovaris M Gambini A Gallo A Falini A Ghezzi A Benedetti B. Axonal injury in early multiple sclerosis is irreversible and independent of the short-term disease evolution. Neurology. (2005) 65:1626–30. 10.1212/01.wnl.0000184493.06254.a6 16301492

